# Neuroinflammation in Ischemic Stroke: Focus on MicroRNA-mediated Polarization of Microglia

**DOI:** 10.3389/fnmol.2020.612439

**Published:** 2021-01-07

**Authors:** Lu Lian, Yunsha Zhang, Lu Liu, Liji Yang, Yichen Cai, Junping Zhang, Shixin Xu

**Affiliations:** ^1^Medical Experiment Center, First Teaching Hospital of Tianjin University of Traditional Chinese Medicine, Tianjin, China; ^2^Tianjin Key Laboratory of Translational Research of TCM Prescription and Syndrome, Tianjin, China; ^3^Tianjin University of Traditional Chinese Medicine, Tianjin, China; ^4^School of Integrative Medicine, Tianjin University of Traditional Chinese Medicine, Tianjin, China; ^5^Binhai New Area Hospital of TCM. Tian Jin, Fourth Teaching Hospital of Tianjin University of Traditional Chinese Medicine, Tianjin, China

**Keywords:** microRNAs, microglia, neuroinflammation, ischemic stroke, polarization

## Abstract

Ischemic stroke is one of the most common causes of death and disability worldwide. Neuroinflammation is a major pathological event involved in the process of ischemic injury and repair. In particular, microglia play a dual role in neuroinflammation. During the acute phase of stroke onset, M2 microglia are the dominant phenotype and exert protective effects on neuronal cells, whereas permanent M1 microglia contribute to prolonged inflammation and are detrimental to brain tissue. Emerging evidence indicates that microRNAs (miRNAs) may have regulatory effects on microglia-associated inflammation. Thus, we briefly reviewed the dynamic response of microglia after a stroke and assessed how specific miRNAs affect the behavior of reactive microglia. We concluded that miRNAs may be useful novel therapeutic targets to improve stroke outcomes and modulate neuroinflammation.

## Introduction

Ischemic stroke is one of the most common causes of death and disability worldwide (Chen et al., 2019). The only effective therapeutic approach to salvage the penumbra in the acute phase of stroke is reperfusion therapy (Knecht et al., 2017; Dela Pena et al., 2018). With improved understanding of the mechanism underlying ischemic stroke, researchers have discovered that several pathogenic events can be triggered by ischemic cell stress, such as excitotoxicity (Lai et al., 2014), inflammation, oxidative stress (Li P. et al., 2018), and mitochondrial disturbances (Narne et al., 2017). These stroke-associated pathogenic events can expand the ischemic core and influence the post-stroke recovery as well as the brain regenerative responses (Kriz and Lalancette-Hébert, 2009).

Among the multiple events leading to short-term mortality and long-term disability, post-stroke inflammation plays an important role in brain tissue damage and repair (Tobin et al., 2014; Yong et al., 2019). Glial cells, especially microglia, are primarily involved in the immune response following an ischemic attack and are activated within hours after the ischemic stroke, thereby maintaining the immunologic homeostasis (Iadecola and Anrather, 2011; Macrez et al., 2011). Moreover, reactive microglia play a dual role in ischemic injuries, which is directly related to the microglial phenotypes. More specifically, the pro-inflammatory M1 phenotype exerts harmful effects, while the anti-inflammatory M2 phenotype plays protective role following ischemic attacks (Michell-Robinson et al., 2015). The phenotypic shift between M1 and M2 in microglia is similar to that of macrophages. An imbalance between M1 and M2 microglia can induce prolonged neuroinflammation and worsen the prognosis. Due to the impact of microglial M1/M2 polarization on neuroinflammation, several studies have proposed microglial polarization modification as a potential treatment for ischemic stroke. More importantly, compared to thrombolytic therapies, therapies targeted at neuroinflammation provide a broader treatment window (Tobin et al., 2014; Yong et al., 2019).

Emerging evidence suggests that microRNAs (miRNAs), the small non-coding RNAs of 18–22 nucleotides play essential roles in stroke pathogenesis (Kosik, 2006; Kabekkodu et al., 2018). Following cerebral ischemia, the expression levels of many miRNAs (i.e., miR-126, miR-145, and miR-320) are significantly altered in rat models of middle cerebral artery occlusion (MCAO) as well as in stroke patients (Dewdney et al., 2018). Furthermore, changes in miRNA expression play key roles in several aspects of stroke pathophysiology, such as excitotoxicity, oxidative stress, mitochondrial disturbances, and inflammation as well as in aspects of post-stroke recovery such as neurogenesis and angiogenesis (Majdi et al., 2016; Li G. et al., 2018). Furthermore, certain miRNAs are potential prognostic, diagnostic, and therapeutic biomarkers for stroke (Vijayan and Reddy, 2016; Mirzaei et al., 2018). The modulation of miRNAs contributes to the post-stroke pathology, including neuroinflammation. Neuroinflammation is strongly associated with stroke, occurs within minutes of focal ischemia, and is involved in all stages of the ischemic cascade (Iadecola and Anrather, 2011). Studies have shown that miRNAs are key mediators in the processes of cerebral ischemia and are modulated in stroke-associated immune cells (i.e., microglia, macrophages, astrocytes, neutrophils, innate lymphocytes, and mast cells) (Karthikeyan et al., 2016; Khoshnam et al., 2017; Gaudet et al., 2018). Furthermore, miRNAs regulating microglia-mediated inflammatory events have been investigated (Freilich et al., 2013; Amici et al., 2017; Wang S. W. et al., 2018; Rodríguez-Gómez et al., 2020). For instance, miRNA-155 acts as a proinflammatory mediator in microglia. MiR-155 inhibition suppresses microglia activation and expression of M1-related inflammatory cytokines (Thounaojam et al., 2014; Guo et al., 2019). In contrast, miR-181c inhibits neuroinflammation by suppressing toll-like-receptor (TLR)-4 expression, thereby inhibiting NF-κB activation and production of downstream cytokines (Zhang et al., 2015).

Therefore, this review describes microglial behaviors such as activation and polarization following ischemic stroke as well as the relationship between miRNAs and the microglial behavior. This will provide further insights into the regulatory roles of miRNAs in microglia-associated inflammation. Further, this review will provide a novel perspective for the future basic as well as clinical research and clinical management of ischemic stroke.

## The Behavior of Microglia Following Ischemic Stroke

Microglia, the resident immune cells of the central nervous system (CNS), are highly plastic the primary cells involved in neuroinflammation caused by ischemia (Iadecola and Anrather, 2011; Gaire et al., 2019). Microglia react to CNS insults and change their phenotypes to clear the neuronal damage through phagocytosis and repair (Nimmerjahn et al., 2005; Hanisch and Kettenmann, 2007). Microglia phenotypes change in response to changes in the brain microenvironment. The ratio of pro- and anti-inflammatory microglia plays the pivotal role in determining the course and severity of neuroinflammation (Yenari et al., 2010). Excessive or inappropriate activation of microglia promotes chronic inflammation and leads to neuropathological progression (Hu et al., 2012).

## The Phenotype of Activated Microglia

Reactive microglia can be divided into two phenotypes according to their function: the pro-inflammatory phenotype M1 and the anti-inflammatory phenotype M2. The activation of the M1 or M2 phenotype is closely related to the environment around the microglia at the time of the ischemic stroke onset (Ponomarev et al., 2013). M1 microglia, induced by lipopolysaccharides (LPS) or interferon gamma (INF-γ), enhance the inflammatory response by producing pro-inflammatory mediators (i.e., IFN-γ, interleukin (IL)-1β, tumor necrosis factor (TNF)-α, and IL-6), reactive oxygen species, nitric oxide, proteolytic enzymes (i.e., metalloproteinase 9 and 3), and chemokines (i.e., chemokine interferon-γ inducible protein). The pathological events caused by pro-inflammatory mediators lead to further brain damage and deterioration. In contrast, the M2 microglia stimulated by IL-4 and IL-13, produce anti-inflammatory cytokines (i.e., IL-10, transforming growth factor-beta(TGF-β), IL-4, IL-13, and insulin-like growth factor 1) and participate in the anti-inflammatory responses that repair damaged brain tissue and exert neuroprotective effects following ischemic insults. Furthermore, previous studies have shown that there are multiple subclasses of M2 phenotypes, and exposure to different stimuli may induce different forms of the M2 phenotype (Mantovani et al., 2004). M2a is induced by IL-4 or IL-13 and is involved in regeneration and repair. M2b exerts immunoregulatory effects and is activated by immune complexes such as Fcγ receptors, TLRs, or IL-1R. Chhor et al. (2013) suggested that the pro-inflammatory stimuli, such as LPS, IL-1β, TNF-α, and IFN-γ induced activation of M1 and M2b. M2c represents an acquired-deactivating phenotype produced in response to IL-10, TGF-β, and glucocorticoids (Ransohoff and Perry, 2009; Chhor et al., 2013; Orihuela et al., 2016; Lam et al., 2017). In addition, new signature genes have been reported to distinguish microglia from infiltrating macrophages during neuroinflammation using RNA sequencing (DePaula-Silva et al., 2019). These data showed that *Sall1* and *SLC2a5* are specifically expressed in reactive microglia, *Fcrls* and *Slc2a5* are expressed in hemostatic microglia, while *Ly6c2* and *Ccr2* are expressed in infiltrating macrophages. Moreover, TREM-1 protein was confirmed as an important cell surface marker for infiltrating macrophages at the peak of neuroinflammation (DePaula-Silva et al., 2019). Furthermore, Schmid et al. (2009) investigated the differentially expressed genes between microglia and macrophages and identified 15 genes including *Loc620695, GPR84*, and *Sal3* enriched in cultured neonatal microglia compared to peritoneal macrophages. However, only three of these 15 genes (*C1qA, Trem2*, and *CXCL14*) were differentially expressed in the adult microglia isolated from LPS/IFN-γ-injected CNS compared to the infiltrating peripheral macrophages. Similarly, Lund et al. (2006) highlighted that the CNS microenvironment may contribute to the different phenotypes of both CNS-resident microglia as well as CNS-infiltrating macrophages. Certain genes associated with inflammatory responses are differentially expressed in *ex vivo* and *in vitro* cultured microglia. For instance, *CX3CR1* and *P2RY12* are downregulated in microglia cultured *in vitro* (Gosselin et al., 2017). The findings described by Gosselin et al. (2017) indicated the limitations that steer microglial phenotypes *in vitro*. Thus, the M1/M2 phenotype transition may not have the same molecular mechanism *in vitro* as that *in vivo*. In summary, fully polarized M1 and M2 microglia/macrophages in neuroinflammation, both *in vivo* and *in vitro*, represent the extremes of a phenotype continuum. Here, we used the terms M1 and M2 to refer to the two extremes of the microglia/macrophage phenotype spectrum. Due to the dual function of microglia, targeting the M1/M2 polarization is postulated as an important therapeutic strategy for cerebral ischemia. The M1 and M2 phenotype ratio is also considered essential in determining whether the brain suffers from secondary injuries following cerebral ischemia (Iadecola and Anrather, 2011; Patel et al., 2013; Barakat and Redzic, 2016; Wan et al., 2016; Wang et al., 2018a; Gaire et al., 2019).

## Dynamic Changes in Microglial Phenotype Following Ischemic Stroke

Owing to the distinct functional phenotypes of microglia, researchers have attempted to identify methods to amplify their beneficial results while suppressing negative effects (Xia et al., 2015; Wang et al., 2018a). Acute ischemic injury is believed to trigger the induction of the M2 phenotype in resting microglia during the early stage of the ischemic stroke, while the left infarcted tissue, following incomplete treatment, stimulates microglia activation and promotes the M1 phenotype (Hu et al., 2012). Thus, we are interested in the dynamic changes of microglial phenotypes depending on the post-stroke different stimulatory environments.

**Figure 1:**
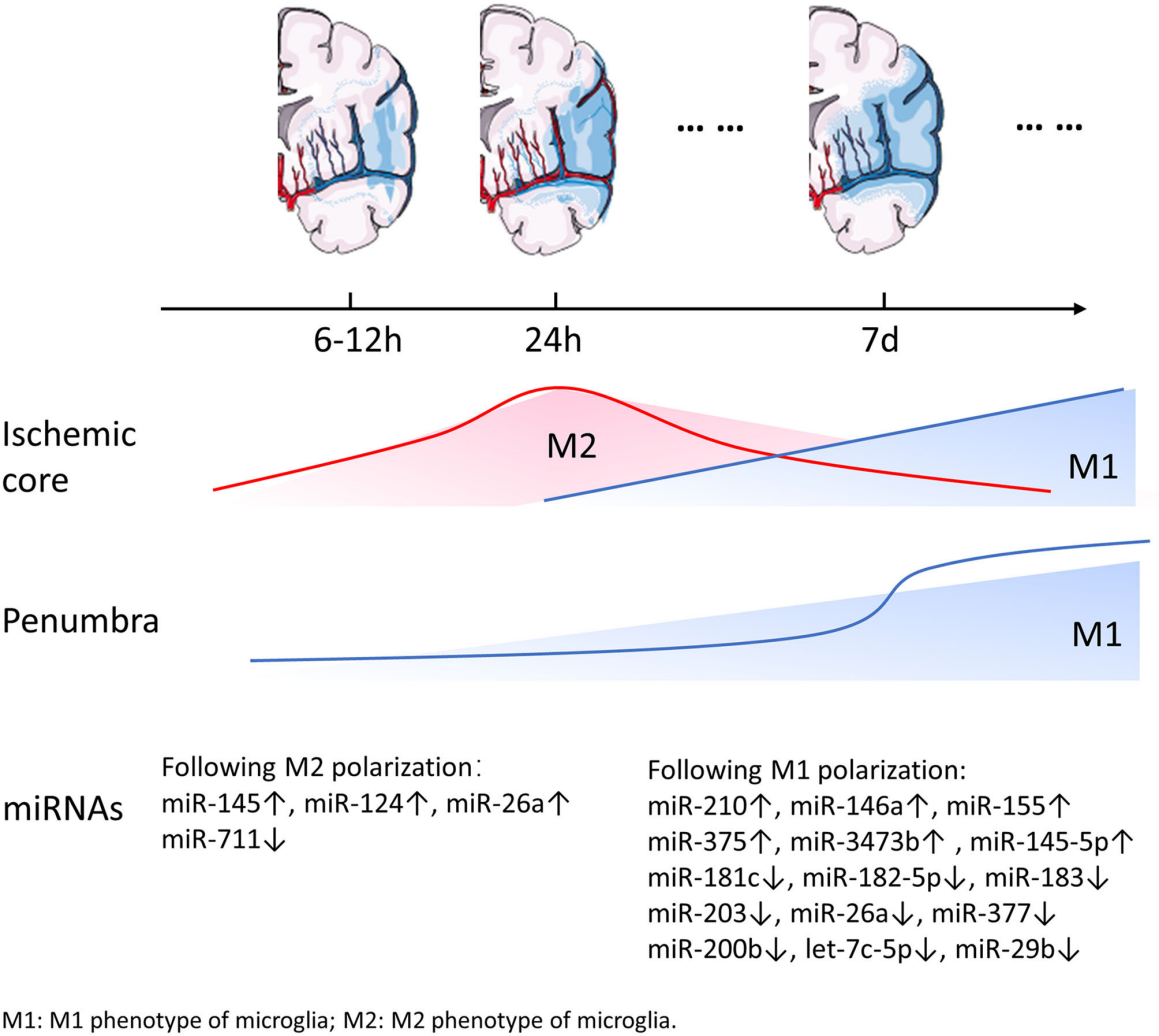
Dynamic changes of miRNAs and microglia following ischemic stroke. Along with the expansion of the ischemic lesions, reactive microglia change dynamically with time and in space. M1 microglia appear to be strongly concentrated in the penumbra and rarely occur in ischemic core within 24 h after stroke. At a later time point M1 microglia appear both in the penumbra and the ischemic core. It is worth noting that the number of M1 microglia increases dramatically 7 d after stroke. M2 microglia are exclusively expressed in the ischemic core in the acute phase of stroke and then gradually decrease. The expression of several miRNAs involved in microglial polarization changes over time.

Ischemic stroke is caused by focal reduction in cerebral blood flow, and result in an ischemic core and the surrounding penumbra. The location of microglia in the ischemic brain affects their activation and cell fate. Microglia respond rapidly to the cell stress signals and migrate to the ischemic area, where they modify their morphology and function within minutes to hours following the onset of ischemia. During expansion of the ischemic lesion, microglia were activated in the surrounding intact area. Resting type ramified microglia were first detected 6 h following MCAO and lasted for 7 d in the ischemic hemisphere. Meanwhile, at 16 and 24 h after MCAO, activated microglia were observed in the infarcted tissue as well as in the area adjacent to infarction (Mabuchi et al., 2000). Simultaneously, peripheral monocytes/macrophages and granulocytes enter the CNS and macrophages cluster at the edge of the infarcted tissue, while granulocytes infiltrate in the center (Mabuchi et al., 2000; del Zoppo, 2009). Several studies have focused on the spatiotemporal features of microglial activation and not the dynamic changes in microglial phenotypes. Recent studies detected M2 microglia/macrophages, which are characterized by Ym1 and CD206, to be exclusively expressed in the ischemic core at 24 h and 7 d after permanent middle cerebral artery occlusion (pMCAO). Furthermore, M2 microglia were the dominant phenotype during the early phase of infarction in the ischemic core. The dominant phenotype gradually switches to the M1 phenotype characterized by CD68 expression within the following 2 weeks (Perego et al., 2011; Hu et al., 2012; Taylor and Sansing, 2013). Perego et al. (2011) provided a detailed description of the microglia phenotype within 2 weeks after stroke. The M1 phenotype surface marker CD68 was expressed 6 h after ischemia and the expression progressively increased during the following 2 weeks, with a dramatic increase on day 7. CD68 appeared to be strongly concentrated in the border zone, and rarely present in the ischemic core during the early phase. However, as the CD68 expression increased gradually, it appeared in the border as well as the core areas. The M2 phenotype markers Ym1 was detected starting from 12 h, reached maximum expression at 24 h post-infarction, and decreased subsequently. The expression of CD206, a M2 phenotype marker, was observed 6 h after pMCAO and reached its peak approximately 24 h following pMCAO. Furthermore, Ym1 and CD206 were expressed exclusively in the ischemic core (Perego et al., 2011). The increase in M1 microglia in the penumbra area may prevent the expansion of the ischemic lesion. During later stages, M1 microglia become dominant and release pro-inflammatory factors, thereby exacerbating neuronal death. M2 microglia that accumulate in the ischemic core may play a role in engulfing debris and promoting wound healing. Similar to the data on the surface markers CD68, Ym1, and CD206, other studies have shown that the expression of M2 phenotype related genes, including *Arg1, CCL22, Ym1/2, IL-10*, and *TNF-*α, increased progressively within 24 h after MCAO and reached a peak after 3–5 d (Hu et al., 2012) (In contradiction with the previously presented data, this study indicated that the M2 related-gene have a different expression pattern than previously described). The M2 microglia/macrophage gene expression decreased after 1 week followed by a return to the baseline expression at 14 d (Mabuchi et al., 2000; Perego et al., 2011; Hu et al., 2012; Ma et al., 2017). However, other studies have indicated that the expression of M2 phenotype related genes will revert to baseline expression 28 d after the ischemia. Parallel to the change in the expression of M2 phenotype related genes, inflammatory genes (such as *CD11b, iNOS*, and *CD16*) in M1 phenotype of microglia, which were stimulated by the enlarging infarcted tissue, were expressed on day 1 and gradually increased until 14 d after MCAO (Liu et al., 2018). Notably, the real-time PCR results for inflammatory or anti-inflammatory genes suggested that the changes in gene expression showed a similar trend regarding the surface markers of M1 or M2 microglia (see Figure 1 for detailed information).

Understandably, the data obtained from a permanent ischemia model and a transient ischemic stroke attack with reperfusion paradigm may not be the same. Kim and Cho (2016) showed that microglia are present in the injured core only in an event of an ischemic/reperfusion injury. In the case of no reperfusion (i.e., pMCAO), microglia appear in the penumbra and their levels decrease gradually (Kim and Cho, 2016). Thus, this is not in agreement with previous findings of Perego et al. (2011) indicating that reactive microglia appeared in the ischemic core as well as in penumbra regardless of their phenotype.

Additionally, reactive astrocytes began to appear around the infarcted area 24 h after MCAO, followed by a variety of glial cells, including microglia, oligodendrocytes, and astrocytes, which are recruited to the edge of the infarction area at the 7 d to promote the formation of glial scars (Zhou et al., 2018). Collectively, microglia dynamically respond to ischemic injury by first inducing the “helpful” M2 phenotype and then inducing the “harmful” M1 phenotype. The future studies should focus on adjusting the balance between M2 and M1 phenotypes to maintain the inflammatory defense and promote neuroprotection, rather than simply reducing the damage caused by M1-related chronic inflammation.

## miRNAs Are Involved in the Molecular Effects of the Different Microglial Phenotypes

Microglia are the main mediators of neuroinflammation and are involved in the progressive brain damage as well as repair following ischemic stroke (Holtman et al., 2017; Wolf et al., 2017). During the complex pathological processes associated with ischemic stroke, a large number of miRNAs regulate the behavior of microglia (Li S. H. et al., 2015; Xiao et al., 2015; He et al., 2016). Animal as well as cellular models have been developed to simulate human cerebral ischemia reperfusion for investigating how these miRNAs affect the role of M1/M2 phenotypes in the damage and repair of brain cells.

## miRNAs Associated With M1 Microglia

LPS and IFN-γ stimulate microglia to exhibit the M1 phenotype, which is a result of activation of many inflammation-related transcription factors (Chhor et al., 2013; Freilich et al., 2013; Orihuela et al., 2016), such as nuclear factor (NF)-κB, activated protein 1 (AP-1), signal transducers and transcriptional activators (STAT1-4), and interferon regulatory factors (IRF 1, 3, 7, and 8). In addition to the pro-inflammatory transcription factors, overexpression of pro-inflammatory cytokines, including iNOS, IL-1β, IL-6, CXCL1, CCL5, and TNF-α, is the characteristic feature of the M1 phenotype (Chhor et al., 2013). Previous studies assessed the differential expression of miRNAs (such as miR-210, miR-155, and miR-375) in LPS-stimulated cells to get a better understanding on the role of miRNAs in regulating expression of inflammatory transcription factors and pro-inflammatory cytokines (Freilich et al., 2013; Cunha et al., 2016). Similarly, certain miRNAs, including miR-182-5p (Wang et al., 2018a), miR-183 (Xiang et al., 2019), and miR-146a (Chu et al., 2018) were shown to be differentially expressed in the plasma of ischemic stroke patients and in the plasma and brains of mice subjected to experimental stroke. Since miRNAs regulate the inflammatory cascades in microglia after classical activation by modulating inflammation-associated pathways (Chu et al., 2018; Li et al., 2020), miRNAs may be a potential therapeutic target for ischemic stroke (see Table 1 for detailed information).

**Table 1:** Relationship between miRNAs and microglia related neuroinflammation in ischemic stroke.

**miRNAs**	**Expression of inflammatory factors**	**Related pathways**	**Impact of stroke**	**References**
miR-210	TNF-α, IL-6, IL-1β, CCL2, CCL3 (↑)	miR-210/SIRT1/NF-κB	Negative^*^	Lou et al., 2012; Huang et al., 2018; Zhang et al., 2019b; Li et al., 2020
miR-181c	TNF-α (↓)	miR-181c/TLR4/NF-κB	Positive	Zhang et al., 2015; Ma et al., 2016
miR-182-5p	TNF-α, IL-6, IL-1β (↓)	miR-182-5p/TLR4/NF-κB	Positive	Wang et al., 2018a
miR-183	IL-1β, IL-6, TNF-α (↓)	miR-183/NF-κB	Positive	Xiang et al., 2019
miR-146a	TNF-α, IL-1β, IL-6 (↓)	miR-146a/IRAK1/NF-κB	Positive	Chu et al., 2018
miR-203	IL-8, TNF-α (↓)	miR-203/MyD88/NF-κB	Positive	Yang et al., 2015; Zhong et al., 2020
miR-155	TNF-α, IL-6, iNOS (↑)	SOCS-1/SHIP-1/JAK/STAT-1/3	Negative	Cardoso et al., 2012; Pena-Philippides et al., 2016
		C/EBP-β/IL-10/STAT-3		
miR-26a	IL-6 (↓)	miR-26a/ATF2	Positive	Kumar et al., 2015
miR-377	TNF-α, IL-6, COX-2, iNOS (↓)	miR-377/EGR2	Positive	Fan et al., 2018
miR-200b	TNF-α, IL-6, iNOS (↓)	miR-200b/cJun/MAPK	Positive	Jadhav et al., 2014
miR-375	–	NDRG2/miR-375/ Pdk1	–	Tang et al., 2016
miR-3473b	TNF-α, IL-6, iNOS (↓)	miR-3473b/SOCS-3	Positive	Wang et al., 2018b
let-7c-5p	iNOS, COX-2, TNF-α, IL-6 (↓)	miR-let-7c-5p/caspase3	Positive	Ni et al., 2015
miR-29b	IL-1β (↓)	miR-29b/SOCS-1/JAK2/STAT3	Positive	Wang et al., 2019a
miR-145-5p	TNF-α (↑)	miR145-5p/Nurr1/TNF-α	Negative	Qi et al., 2017; Xie et al., 2017
		SNHG14/miR-145-5p/PLA2G4A		

* *MiR-210 plays complex role on stroke. MiR-210 presents a positive effect on neuroinflammation, therefore it is negative with outcome of stroke in Huang et al.'s experiment(Huang et al., 2018). While miR-210 may promote angiogenesis and higher miR-210 level is related to a good outcome in stroke patients (Zeng et al., 2011; Lou et al., 2012; Zhang et al., 2019b)*.

## miRNAs Regulate Inflammation via the NF-κB Pathway

NF-κB is one of the major regulators of the inflammatory response, and the NF-κB pathway is activated by multiple inflammatory signals, leading to the expression of a variety of inflammatory and immune-related genes (Bonizzi and Karin, 2004; Vallabhapurapu and Karin, 2009). Previous studies investigated the molecular mechanism underlying the miRNA-induced regulation of the NF-κB signaling in microglia. Li et al. (2020) determined that inhibition of miR-210 reduced expression of pro-inflammatory cytokines, thereby reducing brain damage and ameliorating behavioral deficits induced by MCAO. Furthermore, miR-210 was shown to play an important role in the regulation of the SIRT1/NF-κB pathway (Li et al., 2020). Other *in vivo* studies further supported the finding that the inhibition of miR-210 suppresses the expression of microglial pro-inflammatory factors (Huang et al., 2018). However, Lou et al. (2012), showed that the upregulation of miR-210 may promote angiogenesis following cerebral ischemia, which is beneficial for patient prognosis. Zhang et al. (2019b) intravenously administered exosomes loaded with miR-210 to the ischemic brain and showed that miR-210 loaded exosomes promote VEGF expression and angiogenesis. Moreover, a higher miR-210 level in patients' blood was associated with a good patient outcome following ischemic stroke (Zeng et al., 2011). However, the results related to the role of miR-210 remain inconsistent and require further validation.

Several miRNAs regulate the expression of M1-related inflammatory factors via the NF-κB pathway. For instance, miR-181c, miR-182-5p, and miR-183 regulate the expression of inflammatory factors by inhibiting the activation of the NF-κB signaling pathway. Previous studies have shown that miR-181c and miR-182-5p downregulate TLR4, thereby inhibiting the NF-κB pathway and reducing the production of pro-inflammatory cytokines (Zhang et al., 2015; Wang et al., 2018a). In addition, the levels of miR-181c, miR-182-5p, and miR-183 are downregulated in oxygen-glucose deprivation (OGD)-activated microglia and in the brain tissue of MCAO rat models, and the administration of these miRNAs exerted a protective effect against cerebral ischemia reperfusion injury (Zhang et al., 2015; Wang et al., 2018a; Xiang et al., 2019). Previous studies have shown that the expression of miR-146a is upregulated following LPS stimulation (Juknat et al., 2019). Furthermore, the inhibition of miR-146a triggers an inflammatory response by promoting the activation of the IRAK1/NF-κB signaling pathway, thereby further enhancing the overproduction of TNF-α, IL-1β, and IL-6. Thus, the upregulation of miR-146a exerts a protective effect (Chu et al., 2018). Furthermore, miR-203 regulates the NF-κB signaling via negative feedback by directly targeting the myeloid differentiation primary response gene 88, which leads to the activation of microglia, and promotes the release of inflammatory factors. Previous studies investigating the roles of miR-203 in rats showed its downregulation in the ischemia/reperfusion brain tissue, and that it can inhibit the inflammatory response as well as improve neurological symptoms and brain function following ischemia (Yang et al., 2015; Zhong et al., 2020).

## miRNAs Regulate Inflammation via Other Pathways

In addition to the differential expression of the miRNAs mentioned earlier, upregulated expression of miR-155 (Freilich et al., 2013), miR-375 (Tang et al., 2016), miR-3473b (Wang X. et al., 2018) and the downregulated expression of miR-26a (Kumar et al., 2015), miR-377 (Fan et al., 2018) and miR-200b (Jadhav et al., 2014) were observed in LPS-stimulated primary microglia, BV2 microglia, and N9 microglia cellular models. In addition to the NF-κB pathway, several other pathways are regulated by miRNAs to regulate expression of inflammatory factors.

In microglia are exposed to LPS, miR-155 was shown to exert a pro-inflammatory effect. It also participated in the inflammatory response regulated by suppressor of cytokine signaling molecules-1 (SOCS-1). Cardoso et al. (2012) showed that miR-155 inhibition induces a neuroprotective effect. However, the regulatory role of miR-155 in microglia is complex. In a recent study, a miR-155 inhibitor was injected for three consecutive days starting 48 h after a distal middle cerebral artery occlusion (dMCAO) and the data was analyzed after 7 d post-dMCAO. The results showed that miR-155 inhibition may deactivate STAT-3 and inhibit the JAK/STAT signaling cascade via its direct targets SOCS-1 and src homology 2 domain-containing inositol 5-phosphatase 1(SHIP-1), thereby suppressing the early transient harmful effects of activated microglia/macrophages after 7 d. Subsequently, 14 d after the dMCAO onset, the inhibition of miR-155 most likely triggered the IL-10/STAT-3-mediated anti-inflammatory response, which may have facilitated the upregulation of CCAAT/enhancer-binding protein beta. Finally, miR-155 inhibition enhanced the protective and reparative role of microglia/macrophages 14 d after dMCAO (Pena-Philippides et al., 2016). Differential regulation of neuroinflammation by miR-155 inhibition indicates the complexity of miRNA-mediated regulation of microglial inflammation following ischemic stroke.

Similar to miR-155, miR-375 plays a complex role in regulating the role of microglia following ischemia. A previous study showed that LPS treatment increased N-myc downstream regulated gene 2 (*NDRG2*) expression in N9 microglia, which positively regulated miR-375. Notably, the IL-4 treatment also suppressed the NDRG2 expression, which suggests that NDRG2 controls miR-375 expression across different environments. However, in contrast to the upregulated expression of miR-375 in LPS-stimulated N9 cells, miR-375 expression decreased after cerebral ischemia reperfusion in rats (Tang et al., 2016). Nevertheless, further research is required to gain a better understanding of the influence of microenvironment on miRNA expression *in vivo* and *in vitro*.

Certain miRNAs, such as miR-26a (Kumar et al., 2015), miR-377 (Fan et al., 2018), miR-200b (Jadhav et al., 2014), miR-3473b (Wang et al., 2018b), miR-101a (Saika et al., 2017), let-7c-5p (Ni et al., 2015), miR-29b (Wang et al., 2019a), and miR-145-5p (Xie et al., 2017) were differentially expressed following ischemia and were shown to work through specific proteins and signaling pathways. Downregulation of miR-26a increased the expression of activating transcription factor 2 (ATF2), which led to increased production of IL-6. However, the increased production of TNF-α was not associated with the expression of ATF2. miR-26a increased the expression of TNF-α through a mechanism that was independent of ATF2 (Kumar et al., 2015). Microglia under OGD or LPS treatment showed reduced expression of miR-377 and increased expression of early growth response 2 gene (EGR2). The changes in miR-377 and EGR2 were also present in rats following MCAO. Additionally, Fan et al. (2018) showed that miR-377 can directly regulate EGR2 expression by suppressing the secretion of inflammatory cytokines following OGD. On the other hand, miR-200b expression decreased in activated BV2 microglia, which negatively regulated the cJun/MAPK pathway; thus suppressing the inflammatory response (Jadhav et al., 2014). miR-3473b was shown to participate in post-stroke neuroinflammation by targeting SOCS3 (Wang et al., 2018b). Similar to the previously mentioned targets of miR-155, both SOCS1 and SOCS3 play a role in inhibiting the JAK/STAT1/3 signaling pathway, while SOCS3 mainly inhibits the IL-6 family of cytokines (Baker et al., 2009). Alternatively, miR-101a can increase the ability of microglia to produce IL-6 and TNF-α by inhibiting mitogen-activated protein kinase phosphatase 1 (Saika et al., 2017). Furthermore, the let-7c-5p expression was shown to be reduced in activated microglia, while let-7c-5p overexpression was shown to attenuate LPS-induced iNOS, COX-2, TNF-α, and IL-6 expression. Additionally, the overexpression of let-7c-5p inhibited caspase 3 expression in microglia. A previous study suggested that let-7c-5p levels were decreased in patients with ischemic stroke and in mouse models of MCAO. Its overexpression inhibited post-ischemic neuroinflammation (Ni et al., 2015). In reactive microglia stimulated by OGD, miR-29b is downregulated. Previous studies have shown that the upregulation of miR-29b in microglia inhibits the JAK-2/STAT3 signaling pathway by promoting the expression of SOCS-1; thus suppressing the production of IL-1β. Furthermore, miR-29b inhibition can regulate microglial-induced apoptosis of hippocampal neurons via the JAK-2/STAT3 pathway (Wang et al., 2019a). In an oxygen-glucose deprivation/reperfusion injury model or MCAO model, miR-145-5p was significantly upregulated in microglia. miR-145-5p decreased nuclear receptor related-1 protein (Nurr1) while increasing the TNF-α expression and promoting TNF-α-induced neuronal injury. Thus, blocking the abnormal activation of miR-145-5p/Nurr1/TNF-α axis signaling might improve the neuroinflammatory outcomes and represent an effective therapeutic strategy (Xie et al., 2017).

The complex interactions of miRNAs throughout the neuroinflammation regulatory network are complemented by their complicated regulatory relationships with other regulatory molecules such as long non-coding RNAs (lncRNAs) (Paraskevopoulou and Hatzigeorgiou, 2016). Interestingly, lncRNA can act as a sponge for miRNAs, which reduces the regulatory effect of miRNAs on mRNA (Paraskevopoulou and Hatzigeorgiou, 2016). Additionally, suppression of miR-145-5p has been proven to reduce the pro-inflammatory response, which indicates that lncRNAs could represent another method that can be used regulate miR-145-5p for therapeutic purposes (i.e., SNHG14). Qi et al. (2017) showed that negative regulation of the SNHG14/miR-145-5p/PLA2G4A axis reduces the production of TNF-α.

## miRNAs Associated With M2 Microglia

IL-4 and IL-13 can induce the M2 phenotype in microglia (Chhor et al., 2013; Hamzei Taj et al., 2018). In previous studies, the anti-inflammatory cytokine IL-4 was the only stimulating factor shown to increase the expression of the M2a repair/regeneration phenotypic marker gene. Following IL-4 stimulation, the microglial phenotype transitioned from M0 to M2, leading to a significant upregulation of miR-145 and miR-124 as well as a significant downregulation of miR-711 (Freilich et al., 2013).

Previous studies have shown activated microglia trigger an inflammatory response that may lead to the production of exosomes containing cytokines or pro-inflammatory miRNAs. LPS-stimulated M1 microglia were shown to release miR-155-containing exosomes; thus, promoting neuroinflammation through exosomal transport (Cunha et al., 2016; Li et al., 2019). On the other hand M2 microglia-derived exosomes containing miR-124 were shown to be absorbed by neurons and further boost neuronal survival by targeting the ubiquitin-specific protease 14 (Song et al., 2019). In addition, IL-4-stimulated microglia was suggested to promote angiogenesis by secreting exosomes containing miR-26a, thereby reducing the damage caused by ischemic stroke (Tian et al., 2019). These studies indicate that M2 microglia play a protective role against ischemic stroke through miRNA-mediated regulation.

## miRNAs Are Key Factors in Microglial Phenotypic Switching

Microglia exhibit microenvironment-dependent phenotypes which play either an anti-inflammatory or pro-inflammatory role following cerebral ischemia/reperfusion. The M2 phenotype has neuroprotective effects and works by promoting brain tissue regeneration and repair after ischemia. On the other hand, the M1 phenotype is associated with the release of inflammatory cytokines that aggravate the detrimental effects of the stroke on the brain tissue. miRNAs play essential roles in microenvironmental regulation; therefore, it is necessary to understand the mechanisms through which miRNAs influence the M1/M2 phenotypic transformation. A better understanding of the role of miRNAs in the M1/M2 transformation might provide a new paradigm for stroke treatment.

## Microglial Activation: M0 to M1 or M0 to M2

Resting microglia are activated by LPS or IL-4 and a pro- or anti-inflammatory phenotype is induced. Several miRNAs have a significant role in regulating microglial activation. In this section, we highlight some interesting miRNAs that are known to participate in microglial activation.

### miR-210

The TLR/NF-κB signaling pathway is known to be associated with inflammatory responses (Chen et al., 2018; Zhao et al., 2018). By stimulating microglia with LPS, it has been shown that TLR4 translocated to the microglial membrane and TLR2 expression was increased. Considering that the translocation of NF-κB into the nucleus and the activation of the inflammatory cascade are associated with M1 polarization, the M1 microglia might be mediated via the TLR2/TLR4/NF-kB signaling cascade (Cunha et al., 2016). Thus, miRNAs which regulate inflammatory cytokine production through the TLR/NF-κB signaling pathway, such as miR-210, might regulate microglial polarization. For instance, Li et al. (2020) showed that miR-210 overexpression in neonatal rat primary microglia let to an increase in M1 marker expression (including CD11b, CD45, CD80, and CD86).

### miR-9

miR-9 has also been shown to play a role in microglial transformation. Primary mouse microglia were transfected with lentiviruses expressing the miR-9 precursor to identify the role of miR-9 in mediating the transition of microglia from resting state to the M1 state. Furthermore, miR-9 was shown to be involved in microglial activation via the MCPIP1/NF-κB signaling pathway (Yao et al., 2014; Liu et al., 2016).

### miR-124

Recent studies have suggested that miR-124 plays an important role in regulating the M2 phenotype. Hamzei Taj et al. (2016b) showed that miR-124 increased the expression of arginase-1 (Arg-1), a surface marker of the M2 microglia/macrophage phenotype, and this change was associated with neuroprotection and functional improvement. Furthermore, administration of miR-124 following stroke lead to a significant increase in the M2 and decrease in the M1 microglia/macrophage phenotype. Previous studies indicated that most of the newly recruited microglia and macrophages in the acute phase of neurological diseases are M2; however, after 1 week the M1 phenotype starts to become dominant (Wang et al., 2013; Kumar et al., 2016). miR-124 extended the M2 phase and shortened the M1 phase in following stroke. Hamzei Taj et al. (2016a) reported that treatment with miR-124 intracranial injections both early and delayed contributed to the M1/M2 balance. However, miR-124 administration achieved a maximum impact before the anti-inflammatory phase of the M2 phenotype became dominant (Hamzei Taj et al., 2016a). While the M1 phenotype was not dominant, the effect of the early miR-124 intervention was shown to play a partial role in changing the patterns of M0 to M1 and M0 to M2. Hence, early miRNA administration following stroke leads to maximal increase in protective M2 microglia and a decrease in the pro-inflammatory M1 microglia.

### miR-145 and miR-155

Ingenuity canonical pathway analysis (IPA) and enrichment analysis suggested that upregulation of miR-155 might contribute to the establishment of the M1 state, while upregulation of miR-145 might contribute to the establishment of the M2a state (Freilich et al., 2013). Furthermore, p53-family proteins are notable molecules that play a crucial role in regulating inflammation (Aloi et al., 2015; Agupitan et al., 2020). Studies have shown that the M1 activation in p53^−/−^ microglia was inhibited, while the expression of genes associated with M2 activation was increased. p53 negatively regulated Twist2 through miR-145 and miR-34a, which modulated c-Maf expression in microglia; thus, inhibiting the anti-inflammatory and tissue-repairing effects of c-Maf. (Jayadev et al., 2011; Su et al., 2014; Aloi et al., 2015).

## Phenotypic Switching Between M1 and M2

Another possible approach can be based on the phenotypic switching between the M1 and M2 when the pro-inflammatory M1 phenotype has already become dominant after the stroke. If such a phenotype conversion can be achieved, it will result in a significant improvement in stroke treatment. To investigate this approach, previous studies used sequential cytokine-stimulated microglia to assess microglia repolarization and cell functions. The microglial stimulation with LPS or IFN-γ combined with TNF-α (L + T or I + T), followed by IL-4 or IL-10 showed that secondary IL-4 or IL-10 treatment generally suppressed the L + T or I + T-induced M1 response and further skewed microglia toward an anti-inflammatory M2a state (Siddiqui et al., 2016; Lively and Schlichter, 2018). The repolarization between M1 and M2a states supports the possibility to reprogram the inflammatory response *in vivo*.

Numerous studies have shown that exosomal release from mesenchymal stem cells represents a new type of cell-to-cell communication pathway, which plays a critical role in signal regulation. These membrane-like structures transmit exogenous functional mRNA and miRNAs to target cells and contribute to the development and treatment of disease (Jaimes et al., 2017; Qing et al., 2018). In this review, we mentioned that microglia play a role in signal transduction by releasing miRNA-containing exosomes to mediate the inflammatory response or additional secondary effects of cells. Similarly, the phenotypic transition between M1 and M2 involves signal transduction as well. Zheng et al. (2019) polarized BV2 cells from M0 to M1 phenotype by incubating them with LPS and treated M1 microglia with exosomes secreted from LPS-stimulated macrophages. By quantifying the expression of iNOS (an M1 marker) and Arg1 (an M2 marker), it has been shown that LPS-stimulated macrophages might skew microglial functional polarity from M1 to M2 phenotype (Zheng et al., 2019). This finding showed that exosomes play a role in microglial phenotype conversion. Furthermore, the relationship between the miR-30d-5p-overexpressing exosomes and microglial phenotype was investigated in another study (Jiang et al., 2018). Exosomes derived from miR-30d-5p-overexpressing cells can more effectively inhibit the expression of inflammatory factors and switch microglial phenotype from OGD-stimulated M1 phenotype into the M2 phenotype. Additionally, miR-30d-5p targeted both Beclin-1 and autophagy-related gene 5 (Atg5), thereby inhibiting autophagy-mediated microglial polarization to M1, and promoting the conversion of M1 to M2 (Jiang et al., 2018).

As one of the mechanisms by which miRNAs modulate M1/M2 phenotypes and the lncRNA pattern was also investigated. Studies have shown that taurine upregulated gene 1 (TUG1) gene knockout can promote microglial M1 to M2 phenotypic transformation, and further studies have shown that this transformation will be reversed when miR-145a-5p was inhibited. Thus, these results suggest that TUG1/miR-145a-5p plays a role in microglial polarization (Wang et al., 2019b). A previous study reported the use of the sponge couple GAS5/miR-146a-5p and used isosteviol sodium to break the sponge pair, thus breaking the negative regulation among GAS5/miR-146a-5p/Notch1, and finally inhibiting the expression of pro-inflammatory cytokines as well as promoting the polarization of M1 to M2 phenotype (Zhang et al., 2019a). This regulatory network is regulated by lncRNAs and provides a sophisticated framework for further research and identify the genes involved in the inflammatory response of microglia and their corresponding regulatory miRNAs.

Certain miRNAs have been shown to regulate the transition from M1 to M2 phenotypes in other neurological diseases (i.e., amyotrophic lateral sclerosis, Japanese encephalitis virus, spinal cord injury, and neuropathic pain), thus providing the possibility of studying miRNAs in stroke-related neuroinflammation (Parisi et al., 2016; Yang et al., 2017; Hazra et al., 2019; Liu W. et al., 2020). For instance, miR-128 overexpression significantly decreased the expression of M1 phenotypic marker CD86 as well as CD32 and increased the expression of the M2 phenotypic markers Arg1 and CD206 after compressive spinal cord injury. Furthermore, miR-128 overexpression led to a decrease in the expression of TNF-α, IL-1β, and IL-6 (Yang et al., 2017).

## miRNA-Based Therapeutics Following Stroke

miRNA-based therapeutics can be divided into two categories: miRNA mimics and miRNA inhibitors. Previous studies have investigated the therapeutic values of certain miRNA mimics and inhibitors following stroke. For example, the inhibition of miR-9 activity via microinjection of lenti-anti-miR-9 into the hippocampus/subtantia nigra pars reticulata of mice followed by LPS injection resulted in a dampened inflammatory response (Åkerblom et al., 2013; Yao et al., 2014). The intracerebral injection of miR-124 in a stroke mouse model modulated the microglia/macrophage activation of the M2 phenotype, thus contributing to the restoration of brain cell function (Hamzei Taj et al., 2016a).

Pharmaceutical therapies with immunomodulatory properties could be beneficial in treating ischemic stroke (Tili and Michaille, 2016; Martinez and Peplow, 2017). Pharmaceutical studies have identified that several immunomodulators, including sulforaphane (Eren et al., 2018), triptolide (Feng et al., 2019), and sulfasalazine (Cetin et al., 2017), differentially regulated microglia-associated miRNAs, thereby regulating the expression of inflammatory factors and the shift in microglial polarization.

Sulforaphane is a molecule that exerts antioxidant, anti-inflammatory, and cytoprotective effects and can cross the blood-brain barrier (Tarozzi et al., 2013). Sulforaphane treatment inhibited the expression of M1-related genes after LPS stimulation and reversed the LPS-induced increase in miR-155 expression and decrease in miR-223 expression. Furthermore, the effect of sulforaphane on miR-223 expression was shown to be nuclear factor erythroid 2-related factor 2-dependent (Brandenburg et al., 2010; Eren et al., 2018). Triptolide, a monomeric component isolated from the Chinese herb *Tripterygium Wilfordii* Hook F, suppressed M1 microglial activation by targeting the miR-155-5p/SHIP1 pathway (Feng et al., 2019). Resveratrol, another representative immunomodulator with an anti-inflammatory role, is capable of regulating the expression of miRNAs underlying inflammation (Tili and Michaille, 2016). For instance, resveratrol can reduce the expression of miR-204 and increase the expression of miR-146a-5p, thereby inhibiting LPS-induced inflammation (Li L. et al., 2015; Ge et al., 2019).

Multiple studies have shown that isosteviol sodium has neuroprotective effects in experimental models (Zhang et al., 2017, 2018). Zhang et al. (2019a) showed that isosteviol sodium disrupted the GAS5/miR-146a-5p sponge and suppressed the expression of Notch1 underlying stroke both *in vivo* and *in vitro*, thus shifting the M1 phenotype to the M2 phenotype and inhibiting the expression of pro-inflammatory cytokines. Sulfasalazine, an immune-modulating drug, was shown to exhibit a protective role following cerebral ischemia/reperfusion injury in rats (Cetin et al., 2017) and block microglia from switching to the M1 phenotype both *in vivo* and *in vitro* by upregulating the expression miR-136-5p (Duan et al., 2018).

## Conclusion and Perspectives

Most studies on microglial phenotypes have focused on the process through which miRNAs reduce pro-inflammatory responses, while less attention has been paid to the anti-inflammatory effects shown by the M2 phenotype. This may be due to the M2 phenotype which is expressed transiently during stroke progression, and thus, does not completely alleviate the consequences of the overall inflammation cascade. On the other hand, although more attention has been directed to the phenotypic transformation, there are fewer studies on the role of miRNAs in this field. Thus, the regulatory function of miRNAs in the inflammatory network requires further research. It can be hypothesized that shifting our attention to miRNAs involved in the neuroinflammatory network will provide a new perspective on neuroinflammation regulation. Furthermore, numerous stroke studies do not provide a clear distinction between microglia and macrophages and the difference in the microglial behaviors *in vivo* and *in vitro*. Distinguishing these two cell types and clarifying the effect of the microenvironment on microglial phenotypes might bring new breakthroughs in neuroinflammation regulation.

Microglia play a key role following stroke, and the M1 phenotype typically releases pro-inflammatory mediators, while the M2 phenotype suppresses the harmful processes associated with ischemia. Numerous studies have shown that the interaction of various cells (i.e., astrocytes, oligodendrocytes, neurons), specific cytokines, drugs, and other extracellular components can promote microglia phenotype conversion (Kim and Cho, 2016; Pepe et al., 2017; Zhang et al., 2019a; Liu L. R. et al., 2020). Furthermore, a large number of studies have focused on the combined intervention of miRNAs and microglia; however, the role of miRNAs in the phenotypic transformation of microglia might not be limited to reducing the release of pro-inflammatory factors (Kim and Cho, 2016). The expected treatment should be able to balance the M1 and M2 phenotypes as well as inhibit the inflammation (Liu et al., 2018). Therefore, in many studies, the ratio of M1/M2 is used as an indicator of the treatment effect and success.

A better understanding of the effects of miRNAs on microglia-related neuroinflammation might uncover novel therapeutic targets. For instance, modulating the protein expression of key factors associated with specific diseases through miRNA delivery systems might represent a new approach (Zhang et al., 2015). Currently, miRNA-based therapies involve the use of miRNA mimics and miRNA inhibitors (Wahid et al., 2014; Sun et al., 2018). Certain immunomodulators have been shown to regulate microglia neuroinflammation by interfering with miRNAs (Martinez and Peplow, 2017).

## Author Contributions

Conceptualization: LLian, YZ, and SX. Review of literature: LLian, LLiu, LY, and YC. Writing—original draft preparation: LLian. Writing—review and editing: YZ and SX. Supervision: JZ and SX. All authors have read and agreed to the published version of the manuscript.

## Conflict of Interest

The authors declare that the research was conducted in the absence of any commercial or financial relationships that could be construed as a potential conflict of interest.
